# Socioeconomic inequalities in the use of dental health care among the adult population in Serbia

**DOI:** 10.3389/fpubh.2023.1244663

**Published:** 2023-09-18

**Authors:** Snezana Corovic, Katarina Janicijevic, Snezana Radovanovic, Ivana Simic Vukomanovic, Olgica Mihaljevic, Jelena Djordjevic, Milan Djordjic, Dalibor Stajic, Ognjen Djordjevic, Gordana Djordjevic, Jovana Radovanovic, Viktor Selakovic, Zivana Slovic, Vesna Milicic

**Affiliations:** ^1^Faculty of Medical Sciences, University of Kragujevac, Kragujevac, Serbia; ^2^Department of Social Medicine, Faculty of Medical Sciences, University of Kragujevac, Kragujevac, Serbia; ^3^Faculty of Medical Sciences, Center for Harm Reduction of Biological and Chemical Hazards, University of Kragujevac, Kragujevac, Serbia; ^4^Institute for Public Health, Kragujevac, Serbia; ^5^Department of Pathophysiology, Faculty of Medical Sciences, University of Kragujevac, Kragujevac, Serbia; ^6^Department of Communication Skills, Ethics and Psychology, Faculty of Medical Sciences, University of Kragujevac, Kragujevac, Serbia; ^7^Department of Hygiene and Ecology, Faculty of Medical Sciences, University of Kragujevac, Kragujevac, Serbia; ^8^Department of Epidemiology, Faculty of Medical Sciences, University of Kragujevac, Kragujevac, Serbia; ^9^Department of Forensic Medicine, Faculty of Medical Sciences, University of Kragujevac, Kragujevac, Serbia; ^10^University Clinical Centre Kragujevac, Forensic Medicine and Toxicology Service, Kragujevac, Serbia; ^11^Department of Dermatovenerology, Faculty of Medical Sciences, University of Kragujevac, Kragujevac, Serbia

**Keywords:** socioeconomic inequalities, dental healthcare, adults, National Health Survey, Serbia

## Abstract

**Objectives:**

The aim of this paper is to assess the association of demografic and socioeconomic determinants with utilization of dental services among Serbian adults.

**Materials and methods:**

The study is a part of the population health research of Serbia, conducted in the period from October to December 2019 by the Institute of Statistics of the Republic of Serbia in cooperation with the Institute of Public Health of Serbia “Dr. Milan JovanovićBatut” and the Ministry of Health of the Republic of Serbia. The research was conducted as a descriptive, cross-sectional analytical study on a representative sample of the population of Serbia. For the purposes of this study, data on the adult population aged 20 years and older were used.

**Results:**

Men were approximately 1.8 times more likely than women to not utilize dental healthcare services (OR = 1.81). The likelihood of not utilizing dental healthcare protection rises with increasing age, reaching its peak within the 65–74 age range (OR = 0.441), after which it declines. Individuals who have experienced marital dissolution due to divorce or the death of a spouse exhibit a higher probability of not utilizing health protection (OR = 1.868). As the level of education and wealth diminishes, the probability of abstaining from health protection increases by 5.8 times among respondents with an elementary school education (OR = 5.852) and 1.7 times among the most economically disadvantaged respondents (OR = 1.745). Regarding inactivity, respondents who are not employed have a 2.6-fold higher likelihood of not utilizing oral health care compared to employed respondents (OR = 2.610).

**Conclusion:**

The results suggest that individual sociodemographic factors influence utilization of dental services by Serbian adults and confirmed the existence of socioeconomic disparities.

## Introduction

According to the World Health Organization (WHO), oral health refers to the condition of the mouth, teeth, and orofacial structures, which enables individuals to carry out fundamental activities such as eating, breathing, and speaking. It also encompasses psychosocial aspects, including self-assurance, overall well-being, and the capability to interact socially and perform professional duties free from pain, discomfort, and embarrassment. Oral health undergoes transformations throughout one’s lifespan and is an inseparable component of general health (1). Oral diseases impact approximately 45% or 3.5 billion individuals worldwide, underscoring the fact that almost half of the global population is affected (2). Over the past three decades, the global number of cases has surged by 1 billion, which serves as a clear indication that numerous individuals lack access to sufficient oral healthcare services. Inequalities in oral health are defined as preventable disparities in oral health status that are deemed unfair and unacceptable (3). When addressing socioeconomic disparities in the utilization of dental healthcare, research has revealed that individuals from socioeconomically vulnerable backgrounds possess a greater need for dental services. Paradoxically, they tend to underutilize these services, thereby further exacerbating existing inequalities (4). The accessibility of oral health services exhibits substantial variation, both within countries and across different nations (5). Disparities in dental attendance also persist among various demographic groups (6). High household income, second and middle household wealth status and access to free public health care are significant predictors in the utilization of dental services (7). The odds of non-utilization of dental services were lower for adults living in cities. Sex, skin color, dental treatment needs, poor socioeconomic characteristics, perceived dental treatment needs, and decayed teeth were also associated with non-utilization of dental services (8, 9).

Also, the distribution of oral health services frequently proves inadequate or inaccessible to marginalized or at-risk segments of society. The distribution of dental healthcare providers predominantly favors urban regions, rendering them inaccessible to a significant portion of the population residing in villages and rural areas. Consequently, individuals often incur substantial time and travel expenses when seeking professional oral healthcare services. Therefore, these disparities in oral health status and utilization of dental care services are not inevitable or coincidental. Inequalities arise due to a complex interplay of interconnected factors, many of which are largely beyond the direct influence of individuals. Consequently, investigating these factors becomes invaluable in developing health policy strategies aimed at reducing disparities and inequality in oral health. Within this framework, the aim of this paper is to assess the association of demografic and socioeconomic determinants with utilization of dental services among Serbian adults.

## Methodology

### Study type

The study is a part of the fourth National Health Survey of the Research of Serbia, conducted in the by the Institute of Statistics of the Republic of Serbia in cooperation with the Institute of Public Health of Serbia “Dr. Milan Jovanović Batut” and the Ministry of Health of the Republic of Serbia in accordance with the recommendations for conducting the European health Interview Survey (10).

### Target population

The primary target population consisted of all persons aged 15 and over living in private (non-institutional) households in the Republic of Serbia, who represent the usual population. Excluded are persons in collective households (student dormitories, dormitories for children and youth with disabilities, homes for socially endangered children, homes for pensioners, the older adult and infirm, homes for adults with disabilities, monasteries, convents, etc.). Stratification was performed according to the type of area (urban and other) and the four regions: Belgrade region, Vojvodina region, Sumadija and Western Serbia region, Southern and Eastern Serbia region.

### Sample type

A stratified two – stage cluster sample was used. Random samples of census. A stratified two districts (group of households) were selected with a probability proportional to their size, in the first stage. A sample of households in each enumeration was chosen with an equal probability in the second stage.

### Sample size and sample allocation

The sample size was calculated on the basis of the requirements for the precision of estimates, to assess the standard error of the indicator “proportion of persons prevented from engaging in daily activities” in accordance with EUROSTAT recommendations for conducting European Health Interview Survey (10). It is planned to obtain statistically reliable estimates at the level of Serbia as a whole, then at the level of four regions: Belgrade region, Vojvodina region, Sumadija and Western Serbia region, Southern and Eastern Serbia region, as well as for the population of urban and other settlements. As a compromise between the required precision of estimates and the cost of conducting the research, a sample size of 6,000 households was determined, in which about 15,000 members are aged 15 and over and about 1,500 children aged from 5 to 14. In calculating the sample size children aged from 5 to 14 years have not been taken into account. It was determined that 10 households should be selected in each census round, taking into account the costs of conducting the survey, as well as the time required to complete the survey in the census round. Reserve households were also provided for each census round in the case that a large number of households in the census round refused to cooperate. Dividing the total number of households by the number of households in the sample per census district, it was calculated that 600 census districts should be selected. Sample allocation by region and type of settlement, is proportional to the number of persons aged 15 and over in these contingents, based on current demographic estimates of 2018. A sample of 5,114 households was realized that counted 15,621 persons, from which 13,589 aged 15 and over and 1,493 children aged 5 to 14. For the purposes of this study, data on the adult population aged 20 years and older were used.

### Sample selection frame and sample selection

The 2011 census of the Republic of Serbia was used as a framework for sampling. Census circles formed for the purposes of conducting the census, where they were defined as primary sample units and were selected from each stratum systematically with a probability of choice proportional to size, and the size measure was the number of households in each census circle based on the 2011 Census. Within each stratum, the census districts are sorted according to the municipality to which they belong and the ordinal number within the municipality. Households within each census round were selected with equal probability (simple random) from the list of households recorded in the 2011 Census.

### Time period of data collection

The survey was conducted during 3 months (October–December) in 2019, in accordance with the recommendations of the European Health Interview Survey the third wave that suggests that the period of field data collection must last at least 3 months, of which at least 1 month must be in September–December, i.e., in autumn (10).

### Ethical and legal aspects

Participants of the research were provided with a written document containing the necessary information about the purpose of the study, the scope of their rights and a phone number meant for additional information or possible complaints. An informed notice was acquired via written signature from every participant that accepted to take part in the study. The anonymity in the study was ensured by not taking data that could identify the participant (in accordance with the Law on Official Statistics, the necessary identification was removed and replaced by a code). Databases are located on servers with special access protection, and the results of the research are published in an aggregated form, which fully secures the secrecy of individual data.

### Data collection

Methods of data collection:

1. Self-completion of the questionnaire by the participant without the participation of the interviewers.

The application of the self-completing questionnaire meant that the participant received a structured questionnaire, instructions and filled it out themselves, without the help of interviewers.

### Research instruments

1. A household info panel, which was used to collect information about all members of the household, i.e., socio-economic characteristics of the household itself;
2. A questionnaire was given to each member of the household aged 5 and over (two versions of this questionnaire were used, one for each adult member of the household ages 15 and older, and another for every child and adolescent aged 5 to 14);
3. A self-completion questionnaire, which was filled in independently by each member of the household aged 15 and over. This type of questionnaire was used due to the sensitivity of the questions concerning the use of alcohol, drugs, sexual activities and other. It was not found suitable to fill in by the face to face method.

### Training process and field work

In order to achieve the appropriate quality of the collected data, high rates of household responses and to ensure the sample is representative, before the start of the field work, a selection of members was made. Proper training was given to the interviewers and guidelines were handed down to the supervisors. Seventy teams were formed for the immediate field realization of the research. Each team consisted of two members – one health worker, i.e., nurse/technician or doctor, and one interviewer, with experience in conducting survey research. Sixteen field supervisors were in charge of supervising and controlling the field work. Each supervisor was on average in charge of supervising four teams. The supervisors responded directly to the coordinators. Eight coordinators were organized in four teams. Each team consisted of one expert, a member of the Research Implementation Team and one IT expert. Each of the team coordinators was in charge of the territory of one region and, on average, took charge over four supervisors, i.e., 16 teams of interviewers.

### Monitoring and control of the research process

In order to inform the participants about the research, as well as to provide all the necessary ethical prerequisites for conducting the research, letters for households (prenotification of first contact) and respondents were prepared, which contained basic information related to the research. In addition, IDs and credentials were made that would be carried by the interviewers during the field research. The control procedure of the whole research process, during all its phases, included sampling control and field work control. Sampling control included monitoring the work of interviewers in the process of selecting households, i.e., the use of surrogate households. Control of field work meant control of completed surveys and the number of appropriate household members to be surveyed. Also, during the survey period, the Statistical Office of the Republic of Serbia conducted a control of work through direct contact of households on 25% of the total sample: 15% of households were contacted by phone, while 10% were contacted through field control, i.e., by visiting the household. The control is implemented to include at least one household visited by each survey team. For the purposes of the control, a special questionnaire was prepared and filled out by supervisors. The results of the control showed that the data collection procedure was completed adequately.

### Respondents’ participation in the research and response rate

A total of 5,114 households and 15,621 respondents were included in the survey. Out of a total of 6,335 contacted households, 5,114 of them agreed to participate in the research. The response rate of the households was 80.7%. Out of a total of 13,589 registered members of households aged 15 and over, 13,178 of them agreed or was able to be surveyed, which gives a response rate of 97.0%.

### Variables measured in the study

For the purposes of this study were used both the independent and dependent variables. The independent variables encompass demographic attributes such as gender, age, marital status, and region, as well as socioeconomic factors like education, employment status, and welfare index. On the other hand, the dependent variable of interest is the utilization of dental health services.

### Statistical methods

All data of interest are presented and analyzed by adequate mathematical-statistical methods appropriate for the data type. *χ*^2^ test was applied to test the difference in the frequency of categorical variables. Logistic regression analysis was applied to examine demografic and socio-economic factors associated with inequalities in utilization of dental health services. All results with the probability that is equal to, or less than 5% (*p* ≤ 0.05) were considered statistically significant. Statistical analysis was performed using a commercial, standard software package SPSS, version 19.0. The Statistical Package for Social Sciences software (SPSS Inc., version 19.0, Chicago, IL).

## Results

A total of 12,439 people aged 20 years and older were surveyed. The mean age of respondents was 52.8 ± 17.7 years, and women were significantly older than men (*t* = −6.765, *p* < 0.001). Most of the respondents were married or living in cohabitation (63.2%) and were from Šumadija and Western Serbia region (32%). 56.4% of respondents had a high school diploma, and women were significantly more likely to have a primary school diploma or lower education compared to men (*p* < 0.001). The highest percentage of respondents belonged to the poor category (40.4%). Almost two-thirds of respondents (61.4%) were unemployed or inactive. Men were significantly more likely to be employed (42.9%) than women (32.2%) (*p* < 0.001). The sociodemographic characteristics of respondents by gender and overall are shown in Table 1.

**Table 1:** Sociodemographic characteristics of the adult population of Serbia.

Variables	Gender				Total		*р*
	Male		Female				
	*n*	%	*n*	%	*n*	%	
**Average age ± SD**	51.7 ± 17.5		53.8 ± 17.8		52.8 ± 17.7		<0.001
**Age**							<0.001
20–29	1,260	20.9	1,149	17.9	2,409	19.4	
30–39	991	16.4	958	15	1949	15.7	
40–49	991	16.4	998	15.6	1989	16.0	
50–59	116	18.7	1,261	19.7	2,387	19.2	
60–69	1,072	17.8	1,225	19.1	2,297	18.5	
70–79	592	9.8	816	12.7	1,408	11.3	
80+							
**Marital status**							<0.001
Never married/unmarried community	3,941	65.3	3,903	61.1	7,844	63.2	
Divorce, separation, death of a partner	1,409	23.4	830	13.0	2,239	18.0	
Marriage/common-law union	671	11.1	1,659	26.0	2,330	18.8	
**Region**							0.108
Region of Vojvodina	1,363	22.6	1,549	24.2	2,912	23.4	
Region of Šumadija and Western Serbia	1,343	22.3	1,450	22.6	2,793	22.5	
Region of Southern and Eastern Serbia	1977	32.8	2000	31.2	3,977	32.0	
Belgrade region	1,349	22.4	1,408	22.0	2,757	22.2	
**Education**							<0.001
Primary and lower school	1,174	19.5	1896	29.6	3,070	24.7	
Secondary school	3,739	62.1	3,270	51.0	7,009	56.4	
College and university	1,112	18.5	1,240	19.4	2,352	18.9	
**Well-being index**							0.334
The poorest	2,414	40.0	2,608	40.7	5,022	40.4	
Middle layer	1,206	20.0	1,319	20.6	2,525	20.3	
The richest layer	2,412	40.0	2,480	38.7	4,892	39.3	
**Employment Status**							<0.001
Unemployed	2,586	42.9	2062	32.2	4,648	37.4	
Inactive	1,168	19.4	1,103	17.2	2,271	18.3	
Employed	2,171	36.0	3,192	49.8	5,636	43.1	

More than two thirds of adults aged reported that they have a chosen dentist (elected doctor) (68.2%). A slightly higher percentage of adults had their chosen dentist in private practice, in relation to the public sector (41.7% versus 26.5%). In terms of the results, it was observed that there is a significant difference in dental healthcare utilization based on gender, with women visiting the dentist more frequently (*p* < 0.001). The number of adults who visit their chosen dentist (elected doctor) reduces with age (*р* < 0.001). The greatest number of visits to the dentist in the past year was recorded in the age group of 20–34 years (29.1%), while the lowest number of visits was recorded in the oldest age group of 75+ years (4.2%). Analysis carried out by marital status, indicates that there is a statistically significant difference in the visits to the dentist depending on the marital status of the patients (*р* < 0.001). The highest percentage of respondents who regularly visit their dentist is in the category of married/common-law (62.0%). Significant differences in visits to a dentist exist in the relation to the regions. So, during the last 12 months, the largest percentage of adults from Shumadia and Western Serbia visited a dentist (34.4%), and the lowest percentage was among the adults from Southern and Eastern Serbia (20.7%). There is a statistically significant difference in the distribution of adults who visit their chosen dentist depending on the level of education (*р* < 0.001). In the adults with no education or with primary education, 12.1% of visited a dentist in the last 12 months, and this is smaller number of visits compared to adults with the secondary(60.1%) and high level of education (27.8%). At the same time, the majority of adults who have never visited a dentist is in the group of adults with the lowest level of education (61.7%), which is 12 times more than the ones that have the highest level of education (5.1%). The population of adults, which according to the index of wellbeing belong to the category of the poorest, visit their dentist in a smaller percentage (32.1%) compared to adults who belong to the richest category of the population (48.9%) (*р* < 0.001). Accordingly, the highest percentage of those who have never visited a dentist is among the poorest adults (56.5%), which is 2 times more than among the adults belonging to the richest population group (26.2%). According to the employment status, there is a statistically significant difference in visits to the dentist (*р* < 0.001). Employed adults usually visit a dentist regularly (50.8%) compared to inactive adults who more often never visit a dentist (59.8%) (Table 2).

**Table 2:** Visits to the dentist in relation to the demographic and socioeconomic characteristics of adult population in Serbia.

Variables	Does not know		Less than 6 months ago		6 to 12 months ago		12 months or more ago		Never		*р*
	*n*	%	*n*	%	*n*	%	*n*	%	*n*	%	
**Gender**											
Male	145	49.7	924	42.5	996	46.3	3,816	50.4	151	59.7	<0.001
Female	147	50.3	1,249	57.5	1,153	53.7	3,756	49.6	102	40.3	
**Age**											
20–34	29	9.9	653	30.1	636	29.6	1,056	13.9	35	13.8	<0.001
35–44	19	6.5	470	21.6	455	21.2	980	12.9	25	9.9	
45–54	36	12.3	386	17.8	362	16.8	1,178	15.6	27	10.7	
55–64	47	16.1	326	15.0	356	16.6	1,614	21.3	44	17.4	
65–74	79	27.1	258	11.9	250	11.6	1,653	21.8	57	22.5	
75+	82	28.1	80	3.7	90	4.2	1,091	14.4	65	25.7	
**Marital status**											
Never married/unmarried community	153	53.1	1,365	62.9	1,354	63.1	4,822	63.8	150	59.3	<0.001
Divorce, separation, death of a partner	35	12.2	554	25.5	539	25.1	1,081	14.3	30	11.9	
Marriage/common-law union	100	34.7	252	11.6	253	11.8	1,652	21.9	73	28.9	
**Region**											
Belgrade	40	13.7	654	30.1	488	22.7	1,678	22.2	52	20.6	<0.001
Vojvodina	65	22.3	500	23.0	478	22.2	1700	22.5	50	19.8	
Šumadija and Western Serbia	140	47.9	547	25.2	739	34.4	2,482	32.8	69	27.3	
Southern and Eastern Serbia	47	16.1	472	21.7	444	20.7	1712	22.6	82	32.4	
**Education**											
Primary and lower	139	48.1	236	10.9	260	12.1	2,279	30.1	156	61.7	<0.001
Secondary	137	47.4	1,260	58.0	1,290	60.1	4,238	56.0	84	33.2	
High	13	4.5	675	31.1	597	27.8	1,054	13.9	13	5.1	
**Wellbeing Index**											
The poorest	166	56.8	697	32.1	739	34.4	3,277	43.3	143	56.5	<0.001
Middle layer	54	18.5	427	19.7	421	19.6	1,580	20.9	43	17.0	
The richest layer	72	24.7	1,049	48.3	989	46.0	2,715	35.9	37	26.5	
**Employment Status**											
Unemployed	42	14.6	1,092	50.8	1,060	50.0	2,407	32.2	47	19.1	<0.001
Inactive	48	16.7	418	19.4	404	19.0	1,349	18.0	52	21.2	
Employed	198	68.8	641	29.8	658	31.0	3,719	49.8	147	59.8	

A univariate analysis revealed several predictors of non-use of dental health services, including male gender, older age, lower educational attainment, lower socioeconomic status, unemployment, and individuals who were widowed or divorced. Specifically, men were approximately 1.8 times more likely than women to not utilize dental healthcare services (OR = 1.81). The likelihood of not utilizing dental healthcare protection rises with increasing age, reaching its peak within the 65–74 age range (OR = 0.441), after which it declines. Individuals who have experienced marital dissolution due to divorce or the death of a spouse exhibit a higher probability of not utilizing health protection (OR = 1.868). As the level of education and wealth diminishes, the probability of abstaining from health protection increases by 5.8 times among respondents with an elementary school education (OR = 5.852) and 1.7 times among the most economically disadvantaged respondents (OR = 1.745). Regarding inactivity, respondents who are not employed have a 2.6-fold higher likelihood of not utilizing oral health care compared to employed respondents (OR = 2.610). The multivariate model demonstrates that the most significant demographic and socioeconomic factors influencing dental healthcare utilization are male gender, being widowed, having a lower level of education, and experiencing financial difficulties (Table 3).

**Table 3:** Odds ratios (OR) and 95% confidence intervals (CI) for the association of sociodemographic characteristics with the use of dental health care in study population.

Variables	Univariate model		Multivariate model	
	OR (95%CI)	*p*	OR (95%CI)	*p*
**Gender**				
Male	1.25 (1.194–1.386)	<0.001	1.516 (1.394–1.649)	<0.001
Female	1		1	
**Age**				
20–29	0.088 (0.068–0.114)	<0.001	0.135 (0.099–0.183)	<0.001
30–39	0.099 (0.076–0.128)	<0.001	0.159 (0.118–0.214)	<0.001
40–49	0.148 (0.115–0.192)	<0.001	0.216 (0.161–0.290)	<0.001
50–59	0.214 (0.165–0.277)	<0.001	0.308 (0.231–0.411)	<0.001
60–69	0.331 (0.255–0.429)	<0.001	0.425 (0.325–0.558)	<0.001
70–79	0.441 (0.335–0.580)	<0.001	0.529 (0.400–0.700)	<0.001
80+	1		1	
**Marital status**				
Never married/unmarried community	0.556 (0.505–0.612)	<0.001	0.983 (0.866–1.116)	0.790
Divorce, separation, death of a partner	1.868 (1.674–2.084)	<0.001	1.151 (1.016–1.303)	0.027
Marriage/common-law union	1		1	
**Region**				
Belgrade	0.81 (1.181–1.060)	<0.003	0.885 (0.785–0.997)	0.044
Vojvodina	1.309 (1.309–1.184)	<0.001	1.016 (0.908–1.136)	0.784
Šumadija and Western Serbia	1.293 (1.293–1.158)	<0.001	0.863 (0.761–0.978)	0.021
Southern and Eastern Serbia	1		1	
**Education**				
Primary and lower	5.852 (5.158–6.640)	<0.001	3.589 (3.107–4.146)	<0.001
Secondary	2.021 (1.838–2.222)	<0.001	1.880 (1.695–2.086)	<0.001
High	1		1	
**Wellbeing Index**				
The poorest	1.745 (1.604–1.898)	<0.001	1.294 (1.168–1.435)	<0.001
Middle layer	1.402 (1.268–1.551)	<0.001	1.192 (1.067–1.331)	0.002
The richest layer	1		1	
**Employment Status**				
Unemployed	1.495 (1.347–1.658)	<0.001	1.139 (1.014–1.128)	0.290
Inactive	2.610 (2.396–2.843)	<0.001	1.079 (0.947–1.229)	0.251
Employed	1		1	

*The reference category is: they use dental health care before 12 months.

## Discussion

Many studies show that inequalities in oral health are higher than those in general health, and that the mouth and teeth diseases are more common among the poor (11). Oral health is an important component of the general health of people and inequalities in oral health are most often associated with certain behaviors related to the health, such as smoking, alcohol consumption, unhealthy diet, inadequate hygiene, while they are partly determined by access to healthy food, dental medications and dental services. Many authors believe that the level of usage of dental services in these inequalities have an important role (12), which has been shown in various studies that suggest a connection between socioeconomic factors and the use of dental health care of people around the world. Socioeconomic status was singled out as an important determinant of access to dental health services (13). Less use of dental care is more common in lower socioeconomic status socio-economic groups, primarily because of financial difficulties (14). Also, low socioeconomic status is often associated with a fear of a dental treatment, due to the lack of information. Unemployment is recognized as the one of the important factors that correlate with no usage of the dental care system (13).

The study of socio-demographic factors associated with barriers to accessibility, availability and acceptability of health care usage, identifies female gender as a significant predictor of disparities in the usage of health care that arise from the dual role of women and their responsibilities at the workplace and at home (15). The higher education level contributes to a clearer perception of the problems of oral health. The data show that people with higher levels of education and higher incomes are more likely to use preventive dental health services and have less dental diseases than osobe with low education and low income (16), which is also shown by the results our research. Among the population of rural areas there is a greater presence of caries, missing teeth and filled teeth compared to urban areas. The higher prevalence of oral diseases in adult who come from rural areas is the result of differences in the usage of dental services, oral hygiene habits, different socioeconomic status, health insurance and the abilities of the usage rate of dental services (17). The low level of education, rural environments, improper fear of the dentist and a dental treatment, low income levels and lack of the knowledge on proper techniques and equipment for maintaining oral hygiene are associated with a reduced degree of awareness on the importance of oral health, inadequate maintenance of oral hygiene, infrequent visits to the dentist and reduced use of dental services in the population (18).

Additionally, previous research has consistently indicated the presence of an inequality that favors the affluent when it comes to accessing oral healthcare. The economically disadvantaged population tends to rely more on public healthcare institutions, whereas those with higher socioeconomic status are inclined to seek services from private facilities. Income-related determinants significantly contribute to the greater utilization of public sector services among the poor, while the wealthier individuals are more likely to utilize private sector services. Consequently, poor oral health often serves as a visible manifestation of social disparities (19).Discrepancies in the provision of oral health care indicate inequitable access, resulting in certain groups being at a disadvantage regarding their oral health. Consequently, addressing these disparities entails ensuring that socially disadvantaged groups have equal opportunities to attain and maintain oral health. The pursuit of fairness in oral health care entails endeavors to eliminate inequalities in the provision of oral health services. This includes equal access to available care based on individual needs, equitable utilization of services based on needs, and the provision of equal quality of care for all individuals. Despite the long-standing recognition of the imperative to address disparities in oral health (20), there remains insufficient public attention given to the crucial role of access to high-quality oral health care for low-income individuals, the uninsured, ethnic minorities, immigrants, and rural populations (21, 22). It is necessary to delve more extensively into disparities across all aspects of oral health care, including resource allocation for oral health protection, utilization of oral health services, quality of oral health care services, oral health care workforce, and financing of oral health care, particularly in terms of the financial burden placed on individuals and households (23–27).

Interventions that target various levels of influence are pivotal and can yield superior efficacy compared to those exclusively focused on a single level (28). Enhancing access to oral health care can be achieved through the promotion of supportive networks within families, friends, and the broader social circle, as well as fostering effective collaboration among healthcare providers. Additionally, raising awareness about the significance of oral health and healthcare is essential for shaping public health policies within the community (14).

A comprehensive approach to promoting oral health is crucial, encompassing all stages of life and emphasizing the adoption of appropriate oral health behaviors. This approach should consider both population-level and individual-level interventions that align with the social and cultural determinants of oral health (29).

The integration of universal strategies aimed at the entire population with targeted interventions tailored to high-risk groups is expected to substantially enhance ongoing endeavors to achieve equity in oral health care. Such programs hold significant promise for advancing equitable access and improving oral health outcomes. Interventions aimed at diminishing disparities in oral health care should go beyond the current efforts and focus on strengthening the linkages between healthcare systems and the communities they serve, particularly at the policy and community level. It is crucial to enhance collaboration, communication, and engagement between healthcare providers, policymakers, and the local community to effectively address and bridge gaps in oral health access and outcomes (30).

## Conclusion

The results suggest that individual sociodemographic factors influence utilization of dental services by Serbian adults. Adults men, from lower education, divorced, inactive, from low socioeconomic background reported not utilization oral health care. This study confirmed the existence of socioeconomic disparities in the utilization of dental services among adults in Serbia. The implementation of educational programs and preventive measures, would contribute to raising awareness about the importance of oral health and increased use of dental services. Actions toward health inequalities need to address socioeconomic factors.

## Data availability statement

The raw data supporting the conclusions of this article will be made available by the authors, without undue reservation.

## Ethics statement

The studies involving humans were approved by Institute of Public Health of Serbia “Dr. Milan Jovanović Batut”. The studies were conducted in accordance with the local legislation and institutional requirements. The participants provided their written informed consent to participate in this study.

## Author contributions

SC, KJ, and ISV design and writing manuscript. OM, SR, JR, and VS conducted the statistical analyses, commented on the manuscript and contributed to the background, and discussion section. JDj, DS, and ZS contributed to investigation. GDj, ODj, MDj, and VM critical revision and approval of the final draft. All authors contributed to the article and approved the submitted version.

## Conflict of interest

The authors declare that the research was conducted in the absence of any commercial or financial relationships that could be construed as a potential conflict of interest.

## Publisher’s note

All claims expressed in this article are solely those of the authors and do not necessarily represent those of their affiliated organizations, or those of the publisher, the editors and the reviewers. Any product that may be evaluated in this article, or claim that may be made by its manufacturer, is not guaranteed or endorsed by the publisher.
